# Inhibition of G1P3 expression found in the differential display study on respiratory syncytial virus infection

**DOI:** 10.1186/1743-422X-5-114

**Published:** 2008-10-06

**Authors:** Dongchi Zhao, Dan Peng, Lei Li, Qiwei Zhang, Chuyu Zhang

**Affiliations:** 1 Pediatrics Department, Zhongnan Hospital of Wuhan University Medical School, Donghu Road 169, Wuhan 430071, PR China; 2 Virology Institute, College of Life Science, Wuhan University, Wuhan 430072, PR China

## Abstract

**Background:**

Respiratory syncytial virus (RSV) is the leading viral pathogen associated with bronchiolitis and lower respiratory tract disease in infants and young children worldwide. The respiratory epithelium is the primary initiator of pulmonary inflammation in RSV infections, which cause significant perturbations of global gene expression controlling multiple cellular processes. In this study, differential display reverse transcription polymerase chain reaction amplification was performed to examine mRNA expression in a human alveolar cell line (SPC-A1) infected with RSV.

**Results:**

Of the 2,500 interpretable bands on denaturing polyacrylamide gels, 40 (1.6%) cDNA bands were differentially regulated by RSV, in which 28 (70%) appeared to be upregulated and another 12 (30%) appeared to be downregulated. Forty of the expressed sequence tags (EST) were isolated, and 20 matched homologs in GenBank. RSV infection upregulated the mRNA expression of chemokines CC and CXC and interfered with type α/β interferon-inducible gene expression by upregulation of MG11 and downregulation of G1P3.

**Conclusion:**

RSV replication could induce widespread changes in gene expression including both positive and negative regulation and play a different role in the down-regulation of IFN-α and up-regulation of IFN-γ inducible gene expression, which suggests that RSV interferes with the innate antiviral response of epithelial cells by multiple mechanisms.

## Background

Respiratory syncytial virus (RSV), a leading cause of epidemic respiratory tract infection in infants, spreads primarily by contact with contaminated secretions and replicates in the nasopharyngeal epithelium [1,2]. The respiratory epithelium is postulated to be a primary initiator of pulmonary inflammation in patients with RSV infections [3]. In general, to establish an infection in host cells successfully, viral entry to host cells results in two sets of events: activation of intracellular signaling pathways to regulate pathogenic gene expression [4,5] and subversion of the host's innate immune response [6,7]. RSV infection does not affect the expression of genes belonging to a single biological pathway but causes significant perturbation of global gene expression controlling multiple cellular processes [5]. RSV replication also induces widespread changes in gene expression for cell-surface receptors, chemokines and cytokines, transcription factors, and cell signal transduction elements [8-10].

One pathway to upregulate chemokine gene expression was identified by the activation of mitogen-activated protein kinase and nuclear factor κB during RSV infection [11,12]. The latter signaling cascade cluster includes chemokines, transcriptional regulators, intracellular proteins regulating translation and proteolysis, and secreted proteins [4,9,13], which influence the onset and severity of asthma. For the successful establishment of infection, RSV has also evolved several strategies to escape host cell antiviral mechanisms. Nonstructural proteins 1 and 2 cooperatively antagonize the antiviral effects of type I interferon (IFN) [14-16]. The G glycoprotein functions as a mimic of the CX3C chemokine [17], and during replication RSV displays a conformationally altered mature envelope that is less susceptible to an anti-F glycoprotein neutralizing antibody response [18]. RSV infection inhibits IFN-α/β signaling by specific suppression of signal transducer and activator of transcription (STAT) 1/2 phosphorylation and the degradation of STAT2 expression, providing a molecular mechanism for viral evasion of host innate immune response [6,19,20]. Thus, RSV infection appears to cause widespread changes in gene expression, and multiple mechanisms are involved in the host innate immune response. Here we analyzed the early response of epithelium to RSV infection using differential display (DD) polymerase chain reaction (PCR) amplification of mRNA. Forty DD expression sequence tags (ESTs) were analyzed, and two IFN-inducible genes, G1P3 and MG11, were examined during RSV infection.

## Results

### RSV induced mRNA differential display in SPC-A1 cells

To obtain the DD profile of SPC-1A cells in the presence or absence of RSV infection, total cellular RNA was extracted at 24 h after viral infection. Using an oligo-(dT) primer with A, C or G at the 3'-terminal position and one of 24 arbitrary primers, 72 PCR reactions were performed and produced c.2, 500 interpretable bands on denaturing polyacrylamide gels. Each primer pair combination PCR reaction was run twice. Of the 2,500 bands surveyed, 40 (1.6%) were differentially regulated by RSV infection and were excised for further investigation. The criteria for defining such a DD band have been described [21,22]: differential display cDNAs modulated by RSV needed to show pronounced differences between treatment groups, consistency between two reactions, overall band intensity, and a size of 50–600 nt. In this subjective assessment, 15 DD cDNAs were the most intense, demonstrating extreme differentiation between treatment groups ("on" vs "off" signals); 18 were intense with modest differentiation and seven were less intense, but showed extreme differentiation between treatment groups. Of these 40 excised cDNA bands, 28 (70%) appeared to be upregulated by RSV infection and another 12 (30%) appeared to be downregulated.

### Characterization of differential display bands

These DD cDNAs were successfully reamplified, sequenced, and identified by BLAST searching . Sequences were compared by BLAST against GenBank  and dbEST  with the DD sequence identities established as the highest scoring annotated cDNA or EST sequences. Two ESTs appeared to encode repetitive elements and one was deleted from this DD profile. Thirty-four ESTs from these 40 sequences had been submitted to dbEST [GenBank: CB238796–CB238829]. Twenty-eight ESTs were upregulated by RSV infection and 12 were downregulated in the same samples. Among the twenty-eight upregulated ESTs group, 16 ESTs matched with known genes in GenBank, five matched with dbEST or hypothetical genes or predicted mRNAs without identified function, and seven were sequences with mismatches in either dbEST or GenBank (Table 1). In the downregulated group, four ESTs had homologs to known genes, four matched to dbEST with a definition of hypothetical genes or predicted mRNAs, and four sequences mismatched either in dbEST or GenBank (Table 2).

**Table 1:** ESTs upregulated by RSV infection

Clone_Id	GenBank_Accn.	Homolog definition	Description of the best hit/UniGene ID
SRA01	CB238796	Zinc finger protein 265	Hs.194718
A1-2-1	Unsubmitted	Chemokine C-X-C	Hs.82407
SRA03	CB238798	DNA/Pantothenate	IMAGE: 3857640/metabolism flavop protein
SRA06	CB238801	IFN-γ induced MG11	Hs.371264
SRA10	CB238805	Clathrin, heavy polypeptide	Hs.187416
SRA11	CB238806	NADH dehydrogenase	Hs.198273
SRA15	CB238810	CD79A binding protein 1	Hs.3631
C19-1	Unsubmitted	Soc-2 suppressor	Hs.104315
C19-2	Unsubmitted	Ras-binding protein	SUR-8 mRNA
G23-1	Unsubmitted	Phospholipase	Phospholipase C, gamma 1 (Plcg1)
SRA24	CB238819	Chemokine CC	Hs.10458
SRA19	CB238814	TAF2G/ESTs contigs	TATA box binding protein
SRA13	CB238808	Glucagons precursor	Hs.20529
SRA20	CB238815	Ribosomal protein L19	Hs.426977
SRA02	CB238797	cDNA clones from Liver	Hs.383374
A20-1	Unsubmitted	HSPC129	HSPC129 homolog
SRA09	CB238804	Hypothetical protein	Hs.272688
SRA14	CB238809	ESTs contigs	LOC146901, predicted mRNA sequence
SRA21	CB238816	ESTs contigs	Esophageal cancer associated protein
SRA16	CB238811	ESTs contigs	Clone RP11-165M1
SRA23	CB238818	ESTs contigs	Clone pac408
SRA22	CB238817	Unclassified	Clone RP11-390B4
SRA04	CB238799	Unclassified	Clone RP11-1429F20
SRA05	CB238800	Unclassified	Clone RP11-95O2
SRA07	CB238802	Unclassified	Clone RP11-132B16
SRA12	CB238807	Unclassified	Clone RP11-543F8
SRA08	CB238803	Unclassified	Unmatched
SRA14	CB238809	Unclassified	Unmatched

Note.

UniGene ID: Unique gene cluster ID

IMAGE: The Integrated Molecular Analysis of Genomes and their Expression

ESTs contigs: Sequences were assembled from EST in silico

Unclassified: cDNA cannot be matched to known genes in GenBank

Unmatched: cDNA has no homologs in either GenBank or dbEST.

**Table 2:** ESTs downregulated by infection

dbEST_Id	Clone_Id	GenBank_Accn.	Homolog definition	Description of the best hit/UniGene ID
16938337	SRA33	CB238828	Interferon-stimulated gene	Interferon alpha-inducible protein (G1P3)
16938326	SRA22	CB238817	NADH	NADH dehydrogenase 3 (MTND3)
No	G2202	Unsubmitted	Cyclin D2	Cyclin D2 (CCND2)
16938333	SRA29	CB238824	Elanogaster	LD44720p
16938336	SRA32	CB238827	Hypothetical gene	AK09149
16938334	SRA30	CB238825	CDNA	Predicted cDNA
No	G2-1	Unsubmitted	CDNA	FLB7715 PRO2051
16938332	SRA28	CB238823	ESTs contigs	Unmatched
16938338	SRA34	CB238829	Unclassified	No homolog
16938329	SRA25	CB238820	Unclassified	No homolog
16938335	SRA31	CB238826	Unclassified	No homolog
16938327	SRA23	CB238818	Unclassified	No homolog

Note.

UniGene ID: Unique gene cluster ID

IMAGE: The Integrated Molecular Analysis of Genomes and their Expression

ESTs contigs: Sequences were assembled from ESTs in silico

Unclassified: cDNA cannot be matched to known genes in GenBank

Unmatched: cDNA has no homologs in either GenBank or dbEST.

### Classification of differential display mRNA functions

Among the 20 cloned ESTs, which were matched to their homologs in GenBank or dbETS, two were genes for the chemokines, CC (Hs.10458) and CXC (Hs. 82407), already confirmed to be associated with responses to RSV infection. Others were genes for the Ras-binding protein, zinc finger protein 265, membrane protein CD79A, metabolism flavoprotein, NADH dehydrogenase, phospholipase, and the IFN-γ-inducing factor MG11, which were all upregulated in SPC-A1 cells infected with RSV. Interestingly, RSV infection upregulated expression of the gene for MG11 but suppressed the gene for the IFN-α inducible protein G1P3. These results suggested that RSV replication could induce widespread changes in gene expression including both positive and negative regulation.

### RSV upregulated MG11 and downregulated G1P3 mRNA expressions

**Figure 1:**
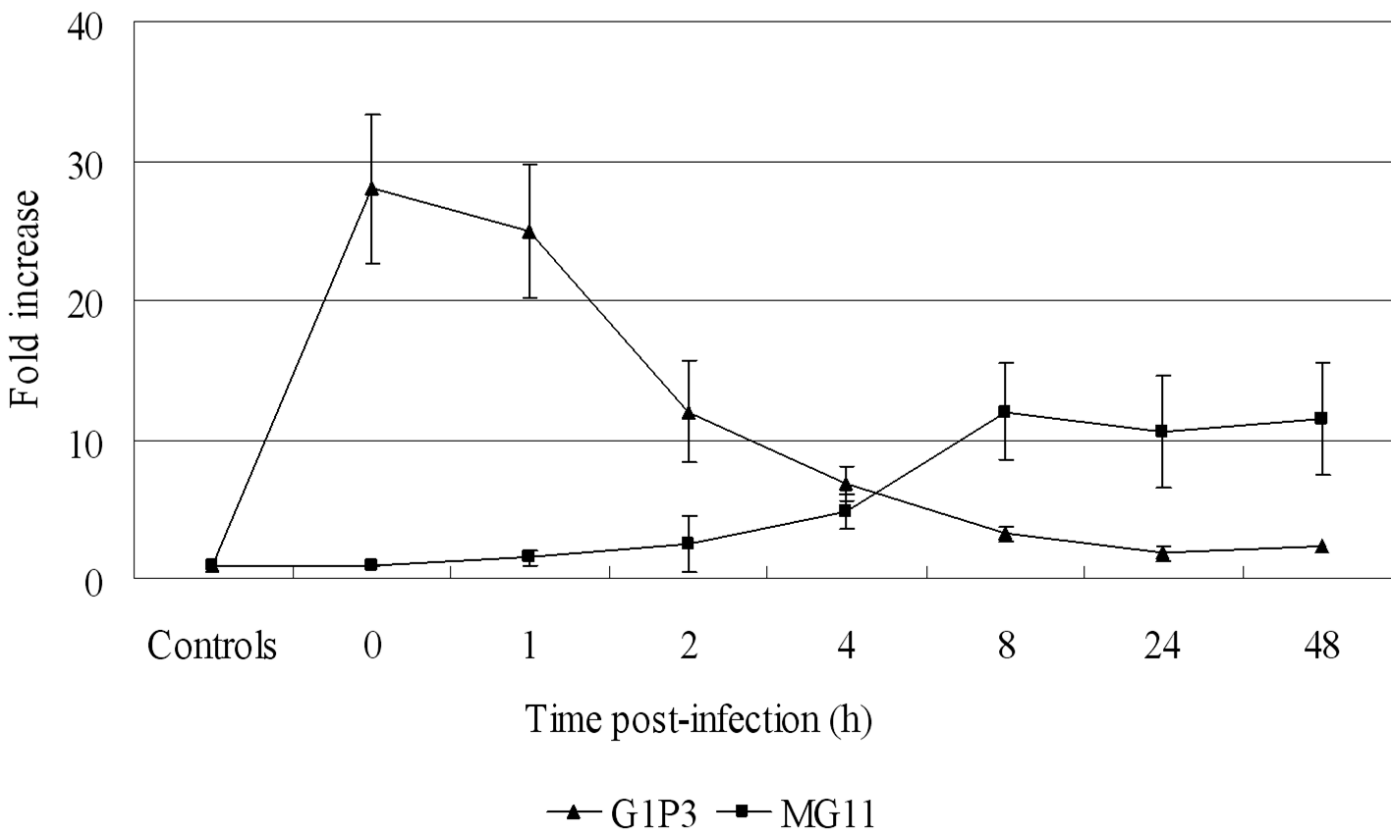
**RSV infection regulates interferon (IFN)-induced gene expression**. SPC-A1 cells were infected with RSV at moi 3, and then INF-a was added into culture at the indicated time points at a final concentration of 1000 U/mL for 30 min. Un-infected cells were treated with IFN-a at time 0, and so on. Total cellular RNA was extracted and G1P3 mRNA was quantified by real-time PCR. To examine MG11, total cellular RNA was extracted at the indicated time points after infection. Data are folds increase compared to un-treated SPC-A1cell controls, and shown as means ± SEs of three independent experiments.

**Figure 2:**
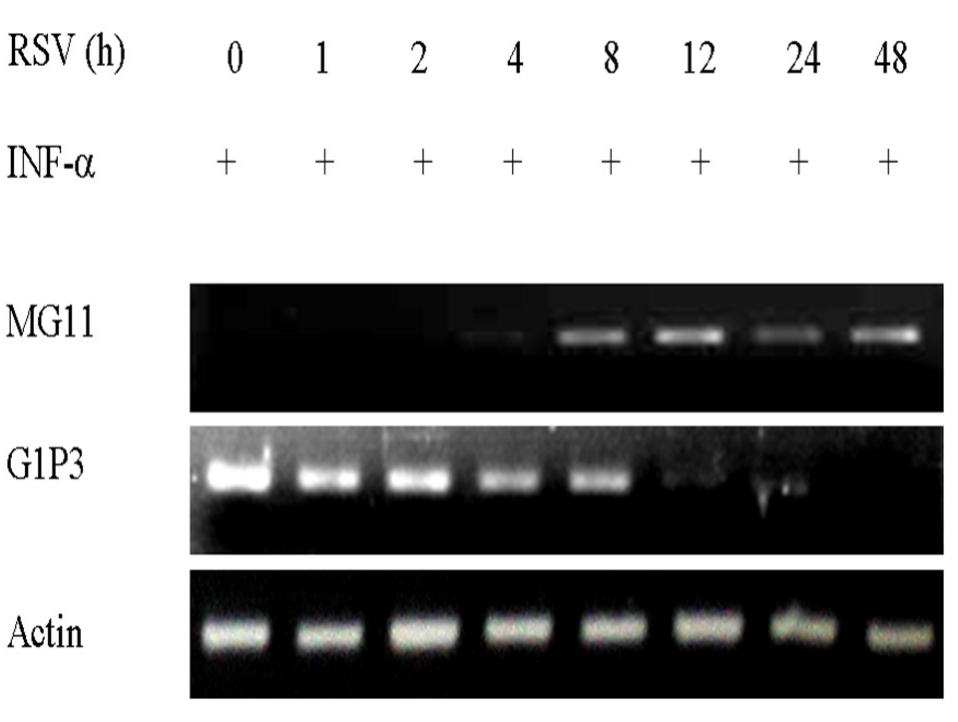
**Agarose gels electrophoreses.** The real-time PCR products were electrophoresed on 2% agarose gels, and shown as one of three different experiments.

To confirm that RSV replication interferes with G1P3 and MG11 mRNA expression in SPC-A1 cells, real-time PCR was performed to quantify mRNA levels after virus infection. To check G1P3 mRNA, SPC-A1 cells were infected with RSV at a multiplicity of infection (MOI) value of 3, and INF-a was added to the culture at final concentration of 1000 U/mL for 30 min. Total RNA was extracted at the indicated time points. RSV inhibited INF-a induced G1P3 expression time-dependently, while it induced MG11 mRNA expression: an IFN-g inducible gene (Fig.1 ). These results suggested that RSV infection plays a different role in the regulation of type a and type g IFN-induced gene expression (Fig. 2).

## Discussion

Differential display is a semiquantitative, RT-PCR based technique that is used to compare mRNAs from two or more conditions of interest [22]. It is usually used to search for specific gene expression patterns associated with diseases and to find novel genes [21]. We tested for differential gene expression in SPC-A1 cells challenged with RSV infection. Our aim was to find novel transcripts modulated by RSV in the early stage of infection. We isolated 40 DD ESTs: 1.6% of c. 2500 bands identified. Sixteen were upregulated and four were downregulated following infection, and these were matched with homologous mRNAs in GenBank. They included IFN-inducible genes and genes for chemokines, membrane molecules, and metabolic factors.

Severe RSV infections involving the lower respiratory tract are primarily seen in young children with naive immune systems or genetic predispositions [1,2]. RSV replication is restricted to airway epithelial cells, where RSV replication induces potent expression of chemokines, so the epithelium is postulated to be a primary initiator of pulmonary inflammation in RSV infections [3]. The presence of eosinophil cationic protein and histamine are correlated with disease severity in the pathology of RSV infections. Here we also found that both chemokines CC and CXC were upregulated during RSV infection in SPC-A1 cells. The mechanisms responsible for recruitment of circulating leukocytes, mononuclear cells, and lymphocytes into the lung because of RSV infection are largely attributed to chemokines [5,23,24]. These are a superfamily of small chemotactic cytokines, which regulate the migration and activation of leukocytes and play a key role in inflammatory and infectious processes of the lung [25,26]. They are divided into functionally distinct groups: three groups of small basic (heparin-binding) proteins, termed the C, CC, and CXC chemokines (based on the number and spacing of highly conserved NH_2_-terminal cysteine residues), and a fourth, distantly related group, the CX3C chemokines, composed of large, membrane-bound glycoproteins attached through a COOH-mucin-like domain.

Other DD mRNAs of interest were for the IFN-induced genes G1P3 and MG11. G1P3, an interferon-stimulated gene (ISG) with a length of 829 bp [27], belongs to the FAM14 family of proteins and has an approximate molecular weight of 13–14 kDa. It has been identified that ectopically expressed G1P3 localized to mitochondria and antagonized TRAIL-mediated mitochondrial potential loss, cytochrome c release, and apoptosis, which contributed the specificity of G1P3 for the intrinsic apoptosis pathway by the direct role of a mitochondria-localized prosurvival ISG in antagonizing the effect of TRAIL[28]. Furthermore, downregulation of G1P3 restored IFN-α2b-induced apoptosis. Curtailing G1P3-mediated anti-apoptotic signals could improve therapies for myeloma or other malignancies. G1P3 was potently induced by IFN-α2b not only myeloma cell lines but also in fresh myeloma cells and resistant to chemotherapy-induced apoptosis. Unlike in cancer cells, the antiapoptotic activity of G1P3 may have a beneficial effect on IFN-mediated antiviral and innate immune responses. During viral infection, delaying early apoptosis through survival factor induction would be a viable cellular strategy to protect surrounding healthy cells from viral infection, enhancing IFN secretion, and overcoming proapoptotic activity of cytokines released into the surrounding milieu. In vitro experiments, the type I IFNs (α/β) induce transcription while type II interferon is a poor inducer of transcription for this gene [29]. IFN-α effectively inhibits hepatitis C virus subgenomic RNA replication and suppresses viral nonstructural protein synthesis. G1P3 enhances IFN-α antiviral efficacy by the activation of STAT3-signaling pathway and intracellular gene activation [30,31]. However, in our experiments, RSV infection appeared to inhibit IFN-α induced G1P3 mRNA, which suggested that virus escaped innate surveillance by subverting IFN-mediated antiviral response.

MG11, encoding a 56-kD protein, was found first in cultured astrocytes stimulated with IFN-γ. There is no evidence to identify this protein's function in host cell antiviral responses [32].

## Conclusion

Our results show RSV replication could induce widespread changes in gene expression including both positive and negative regulation and play a different role in the down-regulation of type α and up-regulation of type γIFN-induced gene expression, which suggests that RSV interferes with the innate antiviral response of epithelial cells by multiple mechanisms.

## Methods

### Virus and cells

The human Long strain of RSV (ATCC, Manassas, VA, USA) was propagated in monolayers of Hep-2 cells grown in Eagle's minimum essential medium Gibco, NY, USA) supplemented with 10% heat-inactivated fetal bovine serum (FBS). At maximum cytopathic effect, the cells were harvested and disrupted by sonication in the same culture medium. The suspension was clarified by centrifugation at 8,000 *g *for 10 min at 4°C and the supernatant was layered on top of a sucrose cushion (30% sucrose in 50 mM Tris buffered-normal saline solution containing 1 mM ethylenediaminetetraacetate [EDTA], pH 7. 5), and further centrifuged at 100,000 *g *for 1 h at 4°C. Pellets containing virus were resuspended in 10 mM phosphate buffered saline containing 15% sucrose and stored in aliquots at -80°C.

SPC-A1 cells (Human typeIIalveolar cell line) were obtained from China Type Culture Collection (CCTCC, Wuhan University, China) and cultured in Dulbecco's Modified Eagle's Medium (DMEM; Life Technologies Gibco BRL, Gaithersburg, MD, USA) supplemented with 10% FBS, 2 mM glutamine, penicillin (100 U/mL), and streptomycin (100 μg/mL) at 37°C under 5% CO_2 _[32,33]. For viral infection, 80% confluent cells were inoculated with RSV at a MIO value of 3. An equivalent amount of sucrose solution was added to the control culture (which received no RSV). The flasks were rocked mechanically for 1 h at 37°C, and then supplemented with 2% FBS+DMEM and incubated at 37°C under 5% CO_2_. To test interferon (IFN)-α inducible gene expression, SPC-A1 cells were infected with RSV at moi 1, and IFN-α (PBL Biomedical Laboratories, Piscataway, NJ, USA) was added to cultures at the indicated times for 30 min to a final concentration of 1000 U/mL.

### Differential display RT-PCR

Differential display RT-PCR was performed as described [21,22]. In brief, cDNA was synthesized from total RNA isolated from SPC-A1 cells using 250 ng 3'-anchored oligo-(dT) 10 μM primers, 3 μg total RNA, 1 μl 10 mM dNTP, 4 μl 5 × First-Strand Buffer, 2 μL 0.1 M DTT, 1 μL ribonuclease inhibitor RNaseOUT (40 U/μL), and 1 μL (200 U) of M-MLV Reverse Transcriptase (Invitrogen Life Technologies, Carlsbad, CA, USA), according to the manufacturer's protocol. cDNA was treated with RNase-free DNase to remove any contaminating genomic DNA. RT-PCR was run with the anchoring primers and one of the 24 random 10-mer primers supplied in the same kit. Amplifications were run for 40 cycles with denaturation at 94°C for 30 sec, annealing at 45°C for 45 sec, and elongation at 72°C for 45 sec with a 10 min extension at 72°C after the last cycle.

### Sodium dodecyl sulfate polyacrylamide gel electrophoresis

After addition of a denaturing loading dye (95% formamide, 0.05% bromophenol blue 0.05% xylene cyanol) and a 2 min, 95°C heat step, PCR products were electrophoresed on 6% denaturing sodium dodecyl sulfate polyacrylamide gels, and developed with 0.1% silver stained according to the protocols of Silver Sequence™ (Promega, Madison, WI, USA) for development and visualization.

### Excision, reamplification, and identification of DD products

Bands that appeared to be differential display were excised from the gels and eluted into 100 μL TE buffer (10 mM Tris/1 mM EDTA) by boiling for 10 min. The eluted DNA samples were then used as templates for PCR reamplification: 1 μL of DD-products were used in a 25 μL PCR reaction containing 2.5 μL of 10 × PCR buffer, 2.5 μL of 10 mM dNTPs, 1 μL of 30 μM downstream primer, 1 μL of 30 μM upstream primer, and 0.5 μL of *Taq *polymerase(Invitrogen Life Technologies, Carlsbad, CA, USA). Cycling conditions were identical to those used for RT-PCR. Reamplified PCR products were electrophoresed on 2% agarose gels, stained with ethidium bromide, excision, and purified with a DNA purification column(E.Z.N.A. TM Ploy Gel DNA Extraction Kit, Omega Bio-Tek, Inc. USA).

### cDNA cloning and sequencing

Differentially expressed cDNA amplicons were subcloned into the pGEM T easy vector ((Promega, Madison, WI, USA) and sequenced using the DYEnamic ET terminator cycle sequencing kit (Amersham Pharmacia Biotech Limited, UK). Sequencing reactions included 0.1 pmol DNA template, 5 pmol universal upstream primer, and 8 μL reagent premix at final volume of 20 μL. Labeling was carried out at 95°C for 20 sec, 50°C for 15 sec, and 60°C for 1 min, for 30 cycles and sequencing was carried out using an ABI PRISM 3100 (Applied Biosystems, Foster City, CA, USA).

### Real-time PCR

Real-time PCR reactions were performed using the protocol of ABI (Applied Biosystems, Foster City, CA, USA). The primer sets were designed for G1P3 (NM_022872), forward: 5'-CCTCGCTGATGAGCTGGTCT-3', reverse: 5'-CTATCGAGATACTTGTGGGTGGC-3', and for MG11 (AK027811), forward: 5'-CTGGAACTCCATCCCGACTA-3', reverse: 5'-GGCAGTAATGCGCCTGTGA-3'. Quantification of cDNA targets was performed using an ABI Prism^® ^7000HT Sequence Detection System (Applied Biosystems), using SDS version 2.1 software. Each reaction contained 10 μL SYBR Green I Master Mix, 1 μL 30 nM forward and reverse primers, and 25 ng cDNA diluted in 9 μL RNase-free water. Thermal cycler conditions were run for 10 min at 95°C, then 40 cycles of 15 sec at 95°C and 1 min at 60°C per cycle using the ABI Prism^® ^7000 Sequence Detection System (Applied Biosystems). All reactions were run in duplicates, and data were normalized to glyceraldehyde-3-phosphate dehydrogenase as an internal control. Real-time PCR data were analyzed using the standard curve method.

### BLAST searching in GenBank and dbEST

Differential display cDNA ESTs were matched in GenBank BLASTN and dbEST. Searches against dbEST were performed to analyze for the abundance of transcripts, to obtain information on possible specificity of mRNA expression, and to identify putative alternative splice forms. Sequences were edited manually by using Sequencher (version 4.14; ) to remove vector sequences and to identify trash sequences, defined as sequences from bacterial DNA, sequences from primer polymers or sequences containing > 5% of ambiguous bases.

## Abbreviations

RSV: Respiratory syncytial virus; DD-RTPCR: Differential display reverse transcription polymerase chain reaction; ESTs: Expression sequence tags.

## Competing interests

The authors declare that they have no competing interests.

## Authors' contributions

DZ developed the study design, laboratory work, participated in data collection, analysis and manuscript writing. DP participated in data collection, laboratory work, data entry and manuscript writing. LL participated in study design, data collection, and laboratory work. QZ developed the data analysis plan and was responsible for data analysis. CZ developed the data analysis plan and manuscript writing. All authors read and approved the final manuscript.
